# Evaluation of NAPLES Prognostic Score to Predict Long-Term Mortality in Patients with Pulmonary Embolism

**DOI:** 10.3390/diagnostics15030315

**Published:** 2025-01-29

**Authors:** Süheyla Kaya, Veysi Tekin

**Affiliations:** 1 Department of Chest Diseases, Selahaddin Eyyübi State Hospital, Diyarbakır 21100, Turkey; aydnn.suheyla@gmail.com; 2 Department of Chest Diseases, Dicle University Hospitals, Diyarbakır 21280, Turkey

**Keywords:** NAPLES, pulmonary embolism, mortality

## Abstract

**Background/Objectives:** Acute pulmonary embolism (APE) is a clinical syndrome characterized by the obstruction of blood flow in the pulmonary artery, whose main pathophysiological features are respiratory and circulatory dysfunction. Acute pulmonary embolism is associated with a high mortality rate. Diagnostic and therapeutic delays can exacerbate mortality and result in prolonged hospitalization. With the increasing understanding that APE is associated with inflammation, various indices based on systemic inflammation have been shown to predict prognosis in patients with APE. The NAPLES Prognostic Score (NPS) is a new scoring system that indicates the inflammatory and nutritional status of the patient based on albumin (ALB) levels, total cholesterol (TC) levels, lymphocyte-to-monocyte ratio (LMR) and neutrophil-to-lymphocyte ratio (NLR). Our study aimed to examinate the effect of NPS on APE prognosis, so the relationship between NPS and APE prognosis was evaluated in our study. In addition, this study seeks to lay the groundwork for further investigations into this association and expand the existing body of knowledge. **Methods:** The clinical data of patients who applied to the Dicle University Faculty of Medicine and were diagnosed with APE between March 2014 and April 2024 were evaluated retrospectively, with 436 patients aged 18 years and over included in the study. Patients were divided into two groups according to NPS. It was statistically investigated whether there was a significant difference in long-term mortality between the two groups. Statistical analyses were performed using Statistical Package for the Social Sciences (SPSS) version 21.0. **Results:** Survival was found to be statistically significantly lower in patients with NPS 3–4 (*p* < 0.05). In the multivariate regression analyses, no statistically significant effect of NPS or other parameters except lactate on 3-month mortality was found (*p* > 0.05). The short-term prognostic value of the NPS has been found to be equivalent to that of the sPESI score. It may be considered that APE patients with high NPS scores should be monitored more frequently. **Conclusions:** Increased NPS was found to be associated with poor APE prognosis in our study.

## 1. Introduction

APE is characterized by the obstruction of blood flow in the pulmonary artery, usually due to a thrombus from a vein in the lower extremity. It is a clinical syndrome whose main pathophysiological features are respiratory and circulatory dysfunction [1]. It may present with classic findings such as shortness of breath and pleuritic chest pain. Less characteristically, it may present with insidious onset of dyspnea lasting days to weeks or syncope with relatively mild respiratory symptoms [2].

The incidence of APE is approximately 60 to 120 per 100,000 people per year. APE, a disease with high mortality, is shown as the third most common cause of death in patients with cardiovascular disease [3].

Although APE is common, it often remains elusive as a diagnosis, so a high clinical suspicion of APE should be maintained for a patient with cardiopulmonary symptoms. The consequences of missing or delaying the diagnosis of APE can be serious. Once suspected, diagnosis is usually straightforward; however, optimal treatment can be difficult. Therefore, early risk stratification is essential to determine appropriate treatment in the management of APE. The Pulmonary Embolism Severity Index (PESI) is frequently used clinically to assess the risk of death in APE patients. However, due to the complexity of risk stratification with the PESI, the need for multiple examinations, its time-consuming nature, the high cost of tests and limitations that prevent rapid assessment of the disease, there is a need to investigate new, cost-effective tools for the prognostic assessment of APE. Biomarkers, as well as clinical scores and imaging, are of great importance in risk stratification, which helps determine the best treatment strategy [2].

With the increasing understanding that APE is associated with inflammation, various indices based on systemic inflammation have been shown to predict prognosis in patients with APE [4]. Various inflammatory or nutritional markers, including serum TC levels, NLR and LMR, have been reported to be associated with the prognosis of APE patients. However, most of the markers included in previous studies were inflammatory or nutritional markers, providing limited information to clinicians and producing controversial results. Therefore, more consistent, comprehensive and validated risk assessment algorithms are needed for APE patients [5,6].

Studies have shown that the NPS has better prognostic predictive value than other inflammatory markers and scoring systems. Recent studies have highlighted that the NPS may have exceptional utility in predicting the prognosis of benign neoplasms. Despite being an easily calculated, simple and reliable prognostic model, NPS has been rarely studied in relation to patients with APE, as evidenced by the limited number of publications on this topic [5].

The NPS is a new scoring system that indicates the inflammatory and nutritional status of the patient based on ALB levels, TC levels, LMR and NLR. It is a simple, reliable and comprehensive prognostic indicator that can be easily calculated. The NPS, which has been investigated in recent years for its relationship with many chronic diseases such as cancer and cardiovascular diseases, can also be an important indicator for APE prognosis. It was thought that new studies should be added to the few studies conducted for this purpose; therefore, in our study, we aimed to evaluate whether there was a relationship between NPS and APE in terms of mortality at 3 months [5,6].

## 2. Materials and Methods

**Figure 1:**
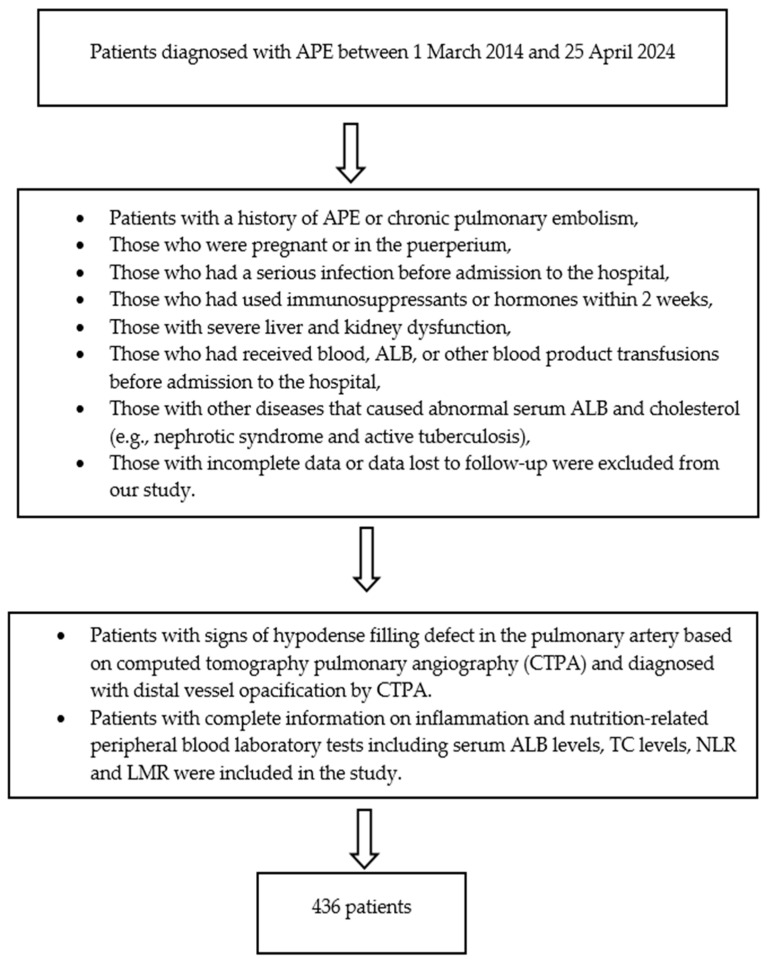
Flowchart of the study.

**Figure 2:**
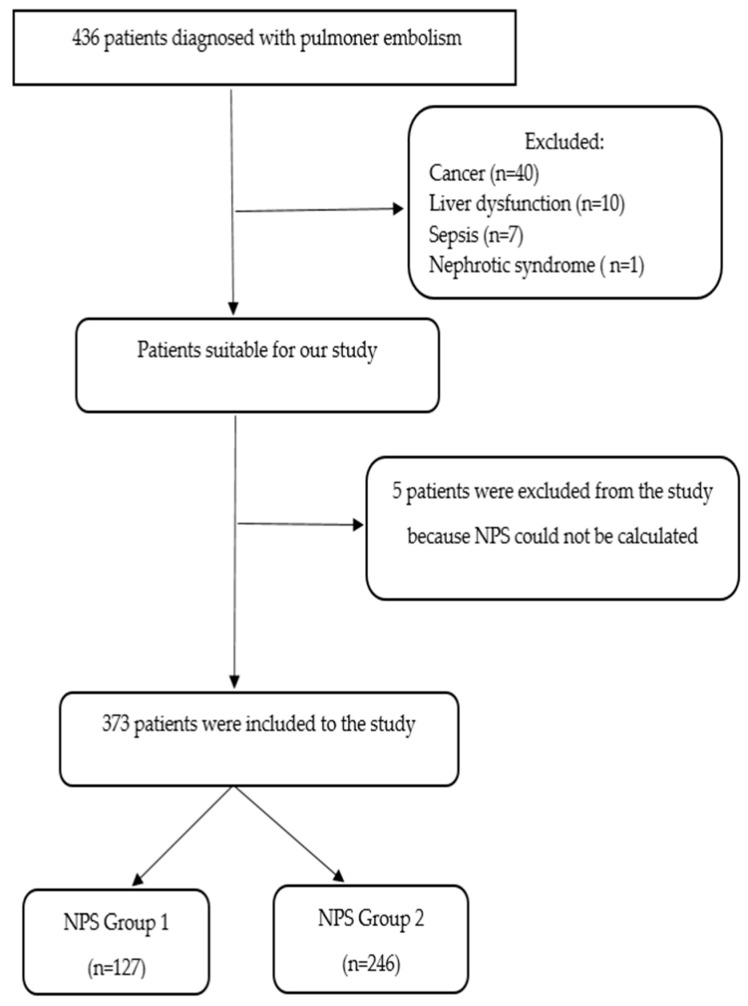
Patient enrollment chart.

Our study was conducted with the approval of the Dicle University Non-Interventional Research Ethics Committee, dated 12 June 2024 and numbered 294. In our study, the clinical data of patients who applied to the Dicle University Faculty of Medicine and were diagnosed with APE between 1 March 2014 and 25 April 2024 were evaluated retrospectively. Patients with signs of hypodense filling defect in the pulmonary artery based on computed tomography pulmonary angiography (CTPA) and diagnosed with distal vessel opacification by CTPA and patients with complete information on inflammation- and nutrition-related peripheral blood laboratory tests including serum ALB levels, TC levels, NLR and LMR were included in the study. Patients with a history of APE or chronic pulmonary embolism; those who were pregnant or in the puerperium; those who had a serious infection before admission to the hospital; those who had used immunosuppressants or hormones within 2 weeks; those with severe liver and kidney dysfunction; those who had received blood, ALB, or other blood product transfusions before admission to the hospital; those with other diseases that caused abnormal serum ALB and TC (e.g., nephrotic syndrome and active tuberculosis) and those with incomplete data or data lost to follow-up were excluded from our study. The demographic, clinical, radiological and laboratory data of the patients were recorded. Findings such as echocardiography findings, liver and kidney function tests, pulse, blood pressure, low/intermediate/high APE, presence of deep vein thrombosis (DVT), clinical findings and intensive care admission were obtained from the hospital information management system (Figure 1 and Figure 2).

All-cause mortality was determined at 30-day and 90-day follow-up from the day of application. The 30-day and 90-day mortality rate was determined from medical records or by contacting patients or relatives regarding survival status or date of death. Patients who died within 30 days of hospital admission were defined as short-term deaths, and patients who died within 90 days were defined as long-term deaths. Patients were classified as survivors and non-survivors based on follow-up results.

Patients were divided into two groups according to NPS scoring. It was statistically investigated whether there was a significant difference in long-term mortality between the two groups.

NPS is calculated using the patient’s preoperative serum ALB, TC, NLR and LMR. The components of the NPS (TC, ALB, NLR and LMR) are clinical biomarkers that are widely used in daily clinical practice and comprehensively reflect the patient’s inflammatory and nutritional status. Therefore, NPS also reflects the patient’s systemic inflammatory response and nutritional status. NPS consists of four parameters: serum ALB, TC, NLR and LMR. In our study, the NPS of all patients was calculated, and the patients were divided into two groups as group 1 and group 2 according to their NPS scores. Group 1 was patients with NPS 0–2, and group 2 was patients with NPS 3–4.

NPS was calculated for all patients at the time of admission, with parameters obtained according to the established method [7]. Patients with NLR ≤ 2.96, LMR ≥ 4.44, ALB ≥ 40 g/L and TC > 180 mg/dL were accepted as 0 points; patients with >2.96, LMR < 4.44, ALB < 40 g/L and TC ≤ 180 mg/dL were accepted as 1 point (Table 1).

Patients with an SI ≥ 1 and right ventricular dysfunction on echocardiogram can receive early reperfusion (thrombolytic) therapy without lengthy imaging studies. SI has been shown to have high sensitivity in identifying subgroups of patients at low risk of mortality (Table 2).

Hemogram parameters were evaluated with Sysmex XN-1000 SA-01 (Kobe, Japan), and biochemical parameters were evaluated with an AU5800 BECKMAN COULTER (Brea, CA, United States) device.

### Statistical Analyses

Statistical analyses were performed using SPSS version 21.0. Descriptive statistics were expressed as number and percentage, mean and standard deviation and median (minimum–maximum).

The conformity of continuous variables to normal distribution was assessed with the Kolmogorov–Smirnov test. The Pearson chi-square test was used to compare categorical variables. The Student’s *t*-test was used to compare continuous variables that were normally distributed between two groups, and the Mann–Whitney U test was used to compare continuous variables that were not normally distributed. The effect of parameters on survival was evaluated by Kaplan–Meier survival analysis and Log-rank test in both groups. The effect of the parameters on NPS was evaluated by multivariate Cox regression analysis. In the statistical analyses in the study, comparisons with a *p* value below 0.05 were considered statistically significant.

## 3. Results

Considering the inclusion and exclusion criteria, 436 patients aged 18 years and over were included in the study.

The mean age, pulse, shock index (SI), modified SI, age-related SI, Aspartate aminotransferase (AST), C-reactive protein (CRP), lactate, right ventricular diameter, right ventricular/left ventricular ratio, pulmonary artery diameter and PAPS values of group 2 were found to be statistically significantly higher than group 1 (*p* < 0.05) (Table 3).

The number of patients with 3-month death, hospital death and heart failure was found to be statistically significantly higher in group 2 (*p* < 0.05) (Table 4).

Presence of DVT, intermediate-low and high patient numbers, chronic obstructive pulmonary disease (COPD), coronary artery disease (CAD), heart failure and immobilization were found to be statistically significantly higher in group 2 (*p* < 0.05) (Table 5).

Survival was found to be statistically significantly lower in patients with NPS 3–4, CRF, ALB < 40 g/L, TC < 180 mg/dL, AST > 50 U/L, CRP ≥ 5 mg/L, high troponin value, lactate ≥ 1.6 mmol/L, sPESI score 1, pulse rate ≥ 100 per min, SBP < 90 mmHg and diastolic blood pressure (DBP) < 60 mmHg (*p* < 0.05) (Table 6a).

**Figure 3:**
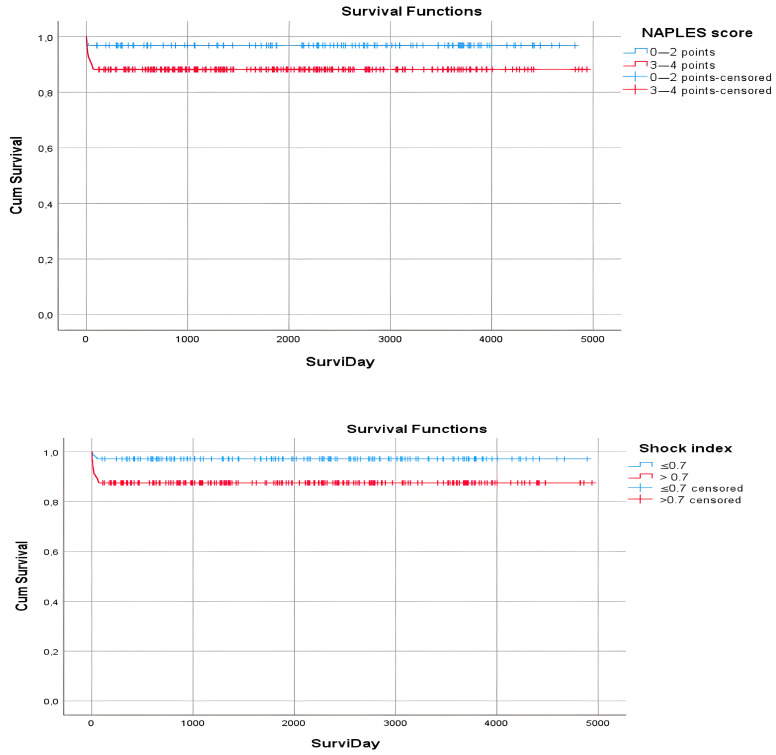

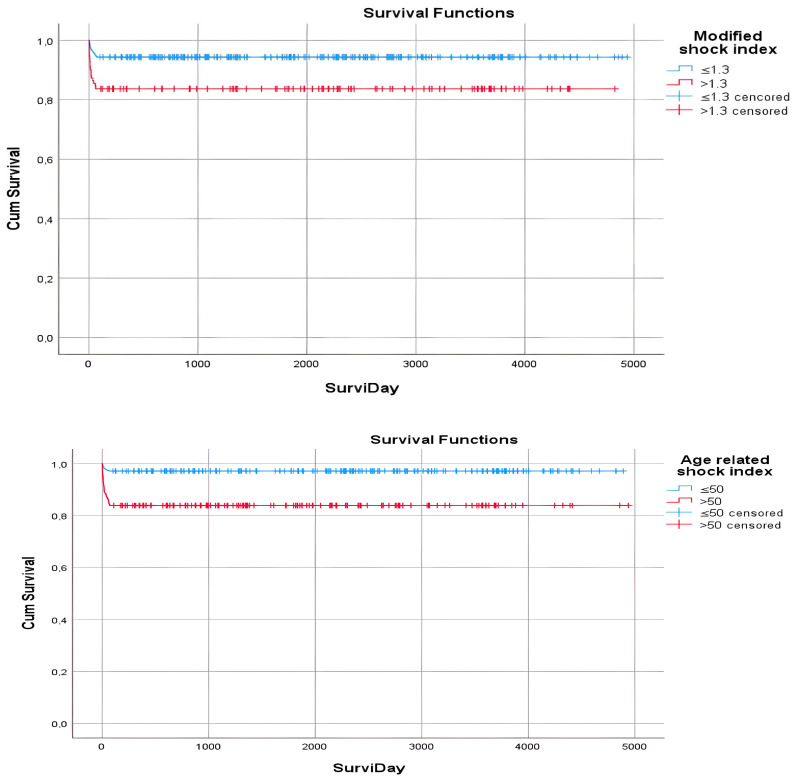
Survival graphs of patients according to NPS and SI parameters.

Survival was found to be statistically significantly lower in patients with an SI greater than 0.7, a modified SI greater than 1.3, an age-related SI greater than 50, a right ventricle value greater than 4, a right ventricle/left ventricle ratio of 0.9 and above, an aortic diameter greater than 3.7, a pulmonary artery diameter greater than 2.1, high APE, intermediate-high APE and those given thrombolytic therapy (*p* < 0.05) (Table 6b and Figure 3).

In the multivariate regression analyses, no statistically significant effect of NPS or other parameters except lactate on 3-month mortality was found (*p* > 0.05) (Table 7).

## 4. Discussion

We evaluated the relationship between NPS and APE prognosis. Survival was found to be statistically significantly lower in patients with NPS 3–4 (*p* < 0.05). In the multivariate regression analyses, no statistically significant effect of NPS or other parameters except lactate on 3-month mortality was found (*p* > 0.05).

Poor immune-nutritional status has been shown to be associated with adverse outcomes in critical illness. The NPS, which has been shown to be associated with prognosis in many serious diseases, especially cancer, is a new scoring based on inflammatory–nutritional indicators [7].

The NPS was first used in patients with colorectal cancer by Galizia et al. in 2017 and was shown to be an independent prognostic indicator for patients undergoing colorectal cancer surgery [7]. In various subsequent studies, it was found that the NPS had prognostic value in many types of cancer like glioblastoma, osteosarcoma, non-small-cell lung, breast, hepatocellular, gastrointestinal, endometrial and pancreatic cancers [8,9,10,11,12,13,14,15,16,17,18].

The prognostic value of the NPS has been investigated in many important diseases other than cancer, such as cardiovascular and cerebrovascular diseases. The effect of NPS on prognosis in spontaneous intracerebral hemorrhage, ST elevation MI (STEMI) and pulmonary arterial hypertension [19,20,21].

An example of studies evaluating the relationship between NPS and prognosis in respiratory system diseases is Zhu et al.’s study in 2023 with 44,601 adult asthma patients. Patients were grouped according to NPS score, and it was shown that the prevalence of asthma and all-cause mortality (cardiovascular diseases, malignancy, respiratory system diseases) were higher in the group with NPS 3–4 [22]. Wu’s study examined the relationship between NPS and lung health in a total of 15,600 participants aged 20 and over. The study stated that NPS is associated with asthma, bronchiolitis and respiratory symptoms; that NPS is negatively associated with lung function; that lung function worsens as NPS increases; and that this score can be used to predict mortality and prognosis in lung-related problems [23].

There are very few studies on the prognosis of NPS in patients with APE. One of these is Pay et al.’s study in 2024 to predict long-term mortality in patients with APE using NPS. In the study, 239 patients diagnosed with APE were divided into two groups according to NPS values, and long-term mortality rates were compared. Mortality was observed in 38 patients at 24-month follow-up. The patients were divided into two groups: group 1, consisting of 215 patients with NPS 0–2; and group 1, consisting of 78 patients with NPS 3–4. Kaplan–Meier survival analysis showed that survival was significantly lower in the second group with high NPS (*p* < 0.001). The study revealed that NPS predicts long-term mortality in APE patients [6]. Another study is Zhu et al.’s study in 2023. The study investigated the value of NPS in predicting 30-day all-cause mortality in APE patients, and 325 APE patients who were hospitalized were divided into three groups according to their NPS values: group 0 (*n* = 131), group 1 (*n* = 153) and group 2 (n = 41). Mortality was observed in 31 patients. In the ROC analysis, it was found that NPS had diagnostic value in predicting 30-day mortality in APE patients (AUC = 0.780, *p* < 0.05, sensitivity = 80.6%, specificity = 72.1). Kaplan–Meier survival analysis showed that group 2 APE patients had the highest risk of all-cause mortality compared to the other two groups. It has been shown that patients with low ALB and TC, high NLR and low LMR have a worse prognosis. The study concluded that the NPS is a new, reliable and multidimensional prognostic scoring system that predicts 30-day all-cause mortality in patients with APE [5]. In our study, in the Kaplan–Meier survival analysis, survival was found to be statistically significantly higher in the patient group with NPS 3–4 compared to the patient group with NPS 1–2 (88.2% and 96.9%, respectively) (*p* < 0.05). In Pay et al.’s study, the mean age was statistically higher, CRP was higher, TC and ALB were lower, NLR was higher, LMR was lower, and D-dimer and Pro-BNP were higher in the group with NPS 3–4 compared to the group with 1–2. Mortality was shown to be statistically significantly higher in the group with NPS 3–4 (*p* < 0.05) [6]. In our study, although similar results were obtained for age, CRP, TC and ALB (*p* < 0.05), no statistically significant difference was found between the two groups for D-dimer and Pro-BNP (*p* > 0.05). In our study, while there was no difference between the two groups in 1-month mortality, 3-month mortality was found to be statistically significantly higher in the group with NPS 3–4 (*p* < 0.05). In addition, the number of patients who died in hospital was found to be statistically significantly higher in this group (*p* < 0.05). Zhu et al.’s study revealed that patients with advanced age, high pulse rate, low systolic blood pressure, low ALB and TC levels, high NLR, low LMR, right heart dilatation, heart failure, malignancy and lower extremity DVT had significantly higher 30-day all-cause mortality (*p* < 0.05) [5]. Similarly, in our study, survival was found to be lower in patients with ALB < 40, TC less than 180, right ventricle/left ventricle value of 0.9 and above, pulse rate of 100 and above, systolic blood pressure less than 90, diastolic blood pressure less than 60 and CRF. In our study, as in Zhu et al.’s study, NLR was found to be statistically significantly higher and LMR lower in the group with NPS 3–4 compared to the group with 1–2 (*p* < 0.05). Although the survival analyses in our study showed that many factors affected survival, no significance was obtained in multiregression analyses except that for lactate. In our study, NPS was not found to have an effect on survival in multivariate regression analyses, and lactate was found to be the only variable that had an effect on survival in these analyses. In the literature, studies on NPS score and APE have yielded significant results in regression analyses, unlike our study. In the regression analysis conducted by Zhu et al., NPS was found to be significantly associated with 30-day mortality [22]. In the study by Pay et al., NPS and sPESI were found to be significantly associated with daily mortality [6].

SI, modified SI and age-related SI are important parameters that have been reported to predict adverse outcomes in patients with different acute cardiovascular conditions. SI is an independent prognostic model of 30-day mortality in patients with PE. In the initial patient management strategy, the SI may identify critically ill patients better than traditional heart rate and blood pressure. It has been used to expedite the triage of patients with suspected APE [24].

In the study by Gökçek et al., the relationship between SI, modified SI and age-related SI and hospital mortality in APE patients was evaluated [25]. It has been reported that the prognostic performance of SI is better than that of other variables in predicting in-hospital mortality in APE patients. The study showed that as SI at admission increased, in-hospital mortality increased. Similar findings were obtained in our study, and survival was observed to decrease as SI, modified SI and age-related SI increased (*p* < 0.05).

In our study, survival was lower in the patients with a higher right ventricle/left ventricle ratio. Zhu et al. showed that right ventricle volume was higher in patients with higher NPS (3–4) (5–6).

Zhu et al. and Pay et al. showed that NLR was higher and LMR was lower in patients with higher NPS (3–4) (*p* < 0.05) (5–6). Neither Pay et al. nor Zhu et al. found significant differences in lactate between NPS 1–2 and NPS 3–4 patients (5–6). In our study, lactate was higher in the NPS 3–4 group.

## 5. Conclusions

It can be said that as NPS increases, the patient’s parameters deteriorate, and, conversely, NPS increases more in patients whose condition is more critical.

Multivariate regression analyses did not show that NPS had an effect on survival. Except for lactate, other parameters were not found to be associated with mortality. However, in survival analyses, it was seen that many parameters, including NPS, had an effect on survival.

The number of studies on this subject in the literature is quite insufficient. In addition, these studies are retrospective studies, and new prospective studies with larger numbers of patients are needed. However, in all these studies, including our study, NPS was found to be associated with APE prognosis. This result is correlated with the aim of our study. If NPS is higher, APE prognosis is worse, so it can be used for APE management.

The limitations of our study can be listed as being a retrospective study and obtaining patient data from the hospital information management system, being a single-center study, having a moderate sample size and not including patients with diseases such as malignancy, chronic liver disease, renal failure and sepsis, which may affect the NPS parameters.

## Figures and Tables

**Table 1:** NPS calculation.

	NPS	
	Cutoff Values	Partial Points
Serum ALB (g/L)	ALB ≥ 40 g/L ALB < 40 g/L	0 point 1 point
TC (mg/dL)	TC > 180 mg/dL TC ≤ 180 mg/dL	0 point 1 point
NLR	NLR ≤ 2.96 NLR > 2.96	0 point 1 point
LMR	LMR ≥ 4.44 LMR < 4.44	0 point 1 point

Group 1: NPS 0–2 points; Group 2: NPS 3–4 points. NPS: NAPLES score, ALB: Albumin, TC: Total cholesterol, NLR: Neutrophil-to-lymphocyte ratio, LMR: Lymphocyte-to-monocyte ratio.

**Table 2:** SI calculation.

Shock Index (SI) Name Variation	Equation	
SI	HR/SBP	
Modified SI (MSI)	HR/MAP	MAP substituted for SBP
Age SI	Age × (HR/SBP)	SI multiplied by patient’s age

SI: Shock Index, HR: Heart rate, SBP: Systolic blood pressure.

**Table 3:** Comparison of some parameters of APE patients between the first group with NPS 0–2 and the second group with NPS 3–4.

	Group 1 NPS 0–2	Group 2 NPS 3–4	*p* Value
Age	58.58 ± 14.95	64.34 ± 16.07	<0.001
Clinical hospital stay	7.32 ± 5.17	7.92 ± 6.36	0.608
Pulse	94.14 ± 17.57	100.79 ± 20.70	0.002
SBP level	122.06 ± 16.57	119.19 ± 19.95	0.116
Average blood pressure level	85.31 ± 11.98	85.47 ± 14.51	0.911
SI	0.79 ± 0.23	0.92 ± 0.66	0.044
Modified SI	1.14 ± 0.33	1.24 ± 0.46	0.026
Age-related SI	47.08 ± 21.74	58.70 ± 42.93	0.004
AST	33.86 ± 82.78	60.63 ± 141.76	<0.001
CRP	29.19 ± 57.67	41.29 ± 62.60	0.011
Thrombocyte	241.35 ± 109.93	241.80 ± 108.02	0.935
Hematocrit	40.50 ± 6.22	37.76 ± 6.86	<0.001
D-dimer	965.23 ± 2021.16	932.28 ± 2961.40	0.170
Lactate	1.66 ± 1.88	1.95 ± 1.75	0.003
Pro-BNP	6681.71 ± 12,700.86	5626.69 ± 8311.57	0.795
Right ventricular diameter	3.59 ± 0.53	3.82 ± 0.61	<0.001
Right ventricle/left ventricle ratio	0.79 ± 0.17	0.83 ± 0.16	0.027
Pulmonary artery diameter	2.06 ± 0.41	2.21 ± 0.54	0.008
PAPS	34.42 ± 17.03	37.59 ± 17.03	0.018

Mann–Whitney U test was applied. SBP: Systolic blood pressure, AST: Aspartate aminotransferase, CRP: C-reactive protein, BNP: Brain natriuretic peptide, PAPS: Pulmonary artery systolic pressure.

**Table 4:** Comparison of some parameters related to morbidity and mortality in APE patients between the first group with NPS of 0–2 and the second group with NPS of 3–4.

	Group 1 NPS 0–2	Group 2 NPS 3–4	*p* Value
3-month mortality			
No	123 (96.8)	217 (88.2)	0.005
Yes	4 (3.1)	29 (11.7)	
Mortality in hospital			
No	123 (96.8)	223 (90.6)	0.029
Yes	4 (3.1)	23 (9.3)	
Location of involvement on CTPA			
Main pulmonary artery	37 (29.1)	83 (33.7)	0.660
Segmental involvement	80 (62.9)	144 (58.5)	
Subsegmental involvement	10 (7.8)	19 (7.7)	
Heart failure			
Yes	8 (6.3)	36 (14.6)	0.018
No	119 (93.7)	210 (85.3)	

Pearson chi-square test was applied. CTPA: Computed tomography pulmonary angiography, CRF: Chronic renal failure.

**Table 5:** Comparison of some APE parameters of APE patients between groups.

	Group 1 NPS 0–2	Group 2 NPS 3–4	*p* Value
DVT existence			
Yes	45 (35.4)	102 (41.4)	0.259
No	82 (64.5)	144 (58.5)	
Doppler not checked	0 (0.0)	0 (0.0)	
Low-risk APE			
Yes	76 (59.8)	87 (35.3)	<0.001
No	51 (40.1)	159 (64.6)	
High-risk APE			
Yes	3 (2.3)	11 (4.4)	0.310
No	124 (97.6)	235 (95.5)	
Intermediate-low-risk APE			
Yes	29 (22.8)	88 (35.7)	0.011
No	98 (77.1)	158 (64.2)	
Intermediate-high-risk APE			
Yes	20 (15.7)	60 (24.3)	0.054
No	107 (84.2)	186 (75.6)	
Thrombolytic administration			
Yes	6 (4.7)	24 (9.7)	0.090
No	121 (95.2)	222 (90.2)	
Additional diseases			
Absent	56 (44.0)	100 (40.6)	0.003
COPD	4 (3.1)	19 (7.7)	
CAD/HF	11 (8.6)	34 (13.8)	
Immobilization	18 (14.1)	48 (19.5)	
Fracture	7 (5.5)	12 (4.8)	
Obesity	8 (6.3)	1 (0.4)	
OC use	3 (2.3)	1 (0.4)	
Others	20 (15.7)	31 (12.6)	

Pearson chi-square test was applied. DVT: Deep vein thrombosis, COPD: Chronic obstructive pulmonary disease, CAD: Coronary artery disease, HF: Heart failure, OC: Oral contraceptive.

**Table 6:** (**a**) Comparison of survival analysis results of some parameters related to APE in APE patients between groups. (**b**) Comparison of survival analysis results of some APE parameters of APE patients between groups.

(**a**)			
	**Total *n*/Censored %**	**Mean**	***p* Value**
NPS			
NPS 0–2	127 (96.9)	4669.4	0.006
NPS 3–4	246 (88.2)	4359.4	
CRF			
Yes	32 (75.0)	3208.8	<0.001
No	341 (92.7)	4578.5	
ALB (g/L)			
≥40 g/L	84 (98.8)	4766.6	0.006
<40 g/L	289 (88.9)	4394.5	
Total cholesterol (mg/dL)			
≥180 mg/dL	176 (96.0)	4482.0	0.002
<180 mg/dL	197 (86.8)	4290.1	
AST (U/L)			
≤50 U/L	309 (93.2)	4560.3	0.001
>50 U/L	64 (81.3)	4015.3	
CRP (mg/L)			
<5 mg/L	117 (95.7)	4650.2	0.036
≥5 mg/L	255 (89.0)	4399.0	
Neutrophil (10^3^/uL)			
<6.96 10^3^/uL	174 (94.3)	4578.9	0.050
≥6.96 10^3^/uL	199 (88.4)	4370.6	
Thrombocyte (10^3^/µL)			
0–149 10^3^/µL	53 (86.8)	4288.2	0.056
150–365 10^3^/µL	286 (93.0)	4550.5	
≥366 10^3^/µL	34 (82.4)	3119.6	
Troponin			
Normal	211 (97.2)	4799.7	<0.001
High	162 (83.3)	3685.2	
Lactate (mmol/L)			
<1.6 mmol/L	213 (97.2)	4800.7	<0.001
≥1.6 mmol/L	160 (83.1)	3246.7	
sPESI score			
0	155 (98.7)	4828.2	<0.001
1	218 (85.8)	4239.6	
Pulse			
<100 per min	207 (94.7)	4632.5	0.006
≥100 per min	166 (86.7)	4286.7	
SBP (mmHg)			
<90 mmHg	22 (68.2)	2198.2	<0.001
≥90 mmHg	351 (92.6)	4574.8	
DBP (mmHg)			
<60 mmHg	84 (84.5)	3788.0	0.012
≥60 mmHg	289 (93.1)	4598.9	
(**b**)			
	**Total *n*/Censored %**	**Mean**	***p* Value**
SI			
≤0.7	141 (97.2)	4752.9	0.002
>0.7	232 (87.5)	4324.1	
Modified SI			
≤1.3	263 (94.3)	4658.7	0.001
>1.3	110 (83.6)	4037.4	
Age-related SI			
≤50	206 (97.1)	4749.1	<0.001
>50	167 (83.8)	4143.9	
Right ventricular value			
≤4 cm	269 (94.4)	4665.1	<0.001
>4 cm	104 (82.7)	3651.7	
Right ventricle/left ventricle ratio			
<0.9 cm	247 (94.3)	4660.6	0.002
≥0.9 cm	126 (84.9)	3749.8	
Aortic diameter value			
≤3.7 cm	288 (93.8)	4631.8	0.001
>3.7 cm	85 (82.4)	3637.2	
Pulmonary artery diameter value			
≤2.1 cm	264 (95.1)	4697.2	<0.001
>2.1 cm	108 (81.5)	3002.2	
Low-risk APE			
Yes	163 (98.2)	4849.1	<0.001
No	210 (85.7)	3790.2	
High-risk APE			
Yes	14 (71.4)	1995.0	0.005
No	359 (91.9)	4541.7	
Intermediate-high-risk APE			
Yes	80 (80.0)	2947.6	<0.001
No	293 (94.2)	4654.0	
Thrombolytic administration status			
Yes	30 (80.0)	2960.6	0.018
No	343 (92.1)	4551.9	

Kaplan–Meier survival analysis and Log-rank test were applied.

**Table 7:** Multivariate Cox regression analysis 3-month mortality results.

	Model 1		Model 2		Model 3		Model 4	
Parameter	HR (%95 CI)	*p* Value	HR (%95 CI)	*p* Value	HR (%95 CI)	*p* Value	HR (%95 CI)	*p* Value
NPS	1.030 (0.243–4.364)	0.968	1.030 (0.244–4.360)	0.968	-	-		-
CRF	2.505 (0.751–8.352)	0.135	2.505 (0.752–8.350)	0.135	2.505 (0.752–8.347)	0.135	2.499 (0.751–8.314)	0.135
ALB	0.194 (0.020–1.926)	0.161	0.194 (0.020–1.904)	0.159	0.195 (0.019–1.857)	0.155	0.192 (0.020–1.805)	0.149
TC	0.659 (0.213–2.037)	0.469	0.659 (0.216–2.008)	0.463	0.664 (0.235–1.883)	0.442	0.669 (0.237–1.885)	0.447
sPESI	0.372 (0.071–1.951)	0.242	0.372 (0.071–1.947)	0.242	0.373 (0.072–1.943)	0.242	0.378 (0.073–1.947)	0.245
Pulse	1.443 (0.359–5.793)	0.605	1.443 (0.360–5.789)	0.605	1.447 (0.363–5.766)	0.600	1.468 (0.375–5.741)	0.581
SBP	0.780 (0.136–4.483)	0.781	0.780 (0.138–4.410)	0.779	0.783 (0.139–4.394)	0.781	0.769 (0.140–4.205)	0.761
DBP	1.093 (0.297–4.025)	0.894	1.093 (0.297–4.014)	0.894	1.093 (0.297–4.014)	0.894	1.082 (0.297–3.940)	0.904
SI	0.735 (0.150–3.604)	0.705	0.735 (0.150–3.597)	0.704	0.735 (0.150–3.597)	0.704	0.738 (0.151–3.608)	0.708
Modified SI	0.688 (0.162–2.910)	0.611	0.687 (0.165–2.868)	0.607	0.686 (0.165–2.852)	0.604	0.678 (0.165–2.786)	0.590
Age-related SI	0.321 (0.084–1.229)	0.097	0.321 (0.084–1.228)	0.097	0.321 (0.084–1.228)	0.097	0.315 (0.085–1.167)	0.084
AST	0.559 (0.207–1.513)	0.253	0.559 (0.209–1.497)	0.247	0.560 (0.211–1.492)	0.246	0.559 (0.210–1.486)	0.244
CRP	0.542 (0.165–1.779)	0.312	0.542 (0.165–1.777)	0.312	0.542 (0.165–1.777)	0.312	0.537 (0.165–1.746)	0.302
Neutrophil	0.550 (0.202–1.499)	0.243	0.550 (0.202–1.497)	0.242	0.552 (0.206–1.482)	0.238	0.551 (0.206–1.477)	0.236
Thrombocyte	0.482 (0.099–2.352)	0.367	0.482 (0.099–2.351)	0.367	0.482 (0.099–2.350)	0.367	0.478 (0.099–2.319)	0.360
Troponin	0.675 (0.148–3.079)	0.612	0.676 (0.151–3.019)	0.608	0.679 (0.155–2.981)	0.608	0.673 (0.154–2.930)	0.598
Lactate	0.242 (0.083–0.705)	0.009	0.242 (0.083–0.705)	0.009	0.243 (0.084–0.705)	0.009	0.241 (0.083–0.699)	0.009
Right vent	0.758 (0.163–3.532)	0.724	0.758 (0.164–3.507)	0.723	0.758 (0.164–3.506)	0.723	0.758 (0.164–3.498)	0.722
Right/left vent	1.376 (0.288–6.576)	0.689	1.377 (0.288–6.575)	0.689	1.380 (0.290–6.564)	0.686	1.364 (0.290–6.405)	0.694
Aortic diameter	0.563 (0.171–1.849)	0.344	0.563 (0.173–1.878)	0.339	0.562 (0.173–1.825)	0.338	0.563 (0.174–1.825)	0.338
Pulmonary artery diameter	0.997 (0.069–14.302)	0.998	-	-	-	-	-	-
Low-risk APE	0.643 (0.098–4.235)	0.646	0.643 (0.098–4.199)	0.645	0.642 (0.098–4.194)	0.644	0.649 (0.100–4.202)	0.650
High-risk APE	1.794 (0.081–39.909)	0.712	1.798 (0.171–18.875)	0.625	1.808 (0.175–18.663)	0.619	1.670 (0.233–11.975)	0.610
Intermediate-high risk	1.577 (0.080–31.126)	0.764	1.582 (0.411–6.091)	0.505	1.593 (0.431–5.888)	0.485	1.561 (0.439–5.544)	0.491
Thrombolytic use	0.903 (0.171–4.782)	0.905	0.904 (0.177–4.602)	0.903	0.902 (0.178–4.576)	0.901	-	-

HR: Hazard ratio.

## Data Availability

Data are available upon reasonable request to the corresponding author.
